# Effects of Immersive and Non-Immersive Virtual Reality on the Static and Dynamic Balance of Stroke Patients: A Systematic Review and Meta-Analysis

**DOI:** 10.3390/jcm10194473

**Published:** 2021-09-28

**Authors:** Aitor Garay-Sánchez, Carmen Suarez-Serrano, Mercedes Ferrando-Margelí, Jose Jesus Jimenez-Rejano, Yolanda Marcén-Román

**Affiliations:** 1 Institute for Health Research Aragón (IIS Aragón), Miguel Servet University Hospital, 50009 Zaragoza, Spain; aitorgaray.fisio@gmail.com; 2 Department of Physiotherapy, University of Seville, 41009 Seville, Spain; jjjimenez@us.es; 3 Department of Human Anatomy and Histology, Institute for Health Research Aragón (IIS Aragón), University of Zaragoza, 50009 Zaragoza, Spain; yomarcen@unizar.es

**Keywords:** stroke, physiotherapy modalities, virtual reality, balance

## Abstract

(1) Background: The development of new technologies means that the use of virtual reality is increasingly being implemented in rehabilitative approaches for adult stroke patients. Objective: To analyze the existing scientific evidence regarding the application of immersive and non-immersive virtual reality in patients following cerebrovascular incidents and their efficacy in achieving dynamic and static balance. (2) Data sources: An electronic search of the databases Medline, Cochrane Library, PEDro, Scopus, and Scielo from January 2010 to December 2020 was carried out using the terms physiotherapy, physical therapy, virtual reality, immersive virtual reality, non-immersive virtual reality, stroke, balance, static balance, and dynamic balance. Selection of studies: Randomized controlled trials in patients older than 18 developed with an adult population (>18 years old) with balance disorders as a consequence of suffering a stroke in the previous six months before therapeutic intervention, including exercises harnessing virtual reality in their interventions and evaluations of balance and published in English or Spanish, were included. A total of two hundred twenty-seven articles were found, ten of which were included for review and of these, nine were included in the subsequent meta-analysis. (3) Data extraction: Two authors selected the studies and extracted their characteristics (participants, interventions, and validation instruments) and results. The methodological quality of the studies was evaluated using the PEDro scale, and the risk of bias was determined using the Cochrane risk-of-bias tool. Data synthesis: Of the selected studies, three did not show significant improvements and seven showed significant improvements in the intervention groups in relation to the variables. (4) Conclusions: Non-immersive virtual reality combined with conventional rehabilitation could be considered as a therapeutic option.

## 1. Introduction

The World Health Organization (WHO) states that stroke represents the leading cause of physical disability in adults. The WHO Program on Cardiovascular Diseases works in the areas of prevention, treatment, and surveillance throughout the world. Its objective is the development of global strategies to reduce morbidity and mortality. It proposes the “development of cost-effective and equitable health care innovations in the field of treatment” [1].

Cerebrovascular accident (CVA) or stroke causes a sudden interruption in physiological brain function that leads to impairments of functional brain networks [2]. In response to the brain damage caused by stroke, changes occur in the structure and function of the central nervous system (CNS); brain networks reorganize their structural and functional anatomy to compensate for both the injury itself and the effects further away [3,4]. Due to neuronal plasticity, damaged brain structures have the capacity for regeneration and for the reorganization of the function of altered neurons [5,6].

The most common disabilities caused by stroke are associated with impaired motor components, hemiparesis, and balance disorders. In addition, these changes compromise the safe walking of patients at home and in the community, increasing the probability of falls [7].

Stroke patients present a lack of control in static and dynamic balance [8]. However, such balance is essential in post-stroke patients for performing activities such as standing, walking, and climbing stairs; therefore, dynamic balance is essential for stroke patients because it is a key determinant of their quality of life [9].

Neuroscientific research has made significant advances in understanding experience-dependent neuroplasticity, and these findings are beginning to be integrated with research on the degenerative and regenerative effects of brain damage [10]. Through sensory integration, the brain organizes somatosensory, visual, and vestibular information and provides crucial information to be used for complex motor skills (maintaining balance, walking, and interacting with the environment) [11,12].

Early physiotherapeutic interventions are fundamental for improving the functional deficit of the post-stroke patient and are aimed at maintaining existing skills, the reacquisition of lost skills, and learning new abilities [13,14]. The facilitation and modulation of neuronal plasticity are necessary to promote motor recovery through interventions with goal-oriented repetitive intensive therapy and with appropriate non-invasive brain stimulation [15].

It is argued that the combination of conventional physiotherapeutic intervention protocols with the use of virtual reality (VR) training systems optimizes results in the recovery of functional deficits in post-stroke patients [16,17,18,19,20,21,22].

Virtual reality can be “non-immersive” or “immersive,” depending on the degree to which the user is isolated from the physical environment when interacting with the virtual environment.

The results of clinical trials [17,23,24,25,26] have found that training based on virtual reality systems is more effective than conventional treatment for relearning and improving balance, mobility, and gait in patients with stroke.

The technology of a variety of non-immersive video game systems developed by the home entertainment industry has become less expensive, which has made this modality more accessible for possible rehabilitation interventions in post-stroke patients [27].

Advances in technology have made it possible to start using immersive virtual reality as a therapeutic approach to improving motor function in stroke. It has demonstrated effectiveness in improving the function of the upper extremities and self-care skills in poststroke patients [28]. In addition, physiotherapeutic interventions based on this type of virtual reality have shown positive effects in patients with spatial negligence after suffering strokes [22].

Therefore, the objective of this review is to analyze the existing scientific evidence regarding the application of immersive and non-immersive virtual reality in patients following cerebrovascular incidents and their efficacy in achieving dynamic and static balance.

## 2. Methodology

### 2.1. Study Design

A systematic review of randomized controlled clinical trials (RCTs) was carried out according to the recommendations established for Systematic Reviews and Meta-analyzes (PRISMA) [29]. This review was registered in the PROSPERO database (CRD42020154930).

### 2.2. Information Sources

An electronic search of the databases Medline, Cochrane Library, PEDro, Scopus, and Scielo for the last ten years was carried out.

The search terms were developed using the PICOS format [30], following the Population, Intervention, Comparator, Results, and Study Design proposed by the York Center for Reviews and Dissemination team.

### 2.3. Search Strategy

The search terms used were:

- “Physiotherapy” or “Physical therapy”;
- “Virtual Reality”;
- “Immersive Virtual Reality”;
- “Non-immersive Virtual Reality”;
- “Stroke”;
- “Balance”;
- “Static Balance”;
- “Dynamic Balance”.

These terms were combined in the different searches through the Boolean operators AND and OR (Table 1).

In Medline, the following filters were used: “Article type” (RCT and clinical trial protocol), “Publication date”, species (humans), and languages (English and Spanish).

In PEDro, we used, in an advanced search, “Neurology” for the subdiscipline, and “clinical trial” for the method.

In Scopus, the filters were used to set the type of document, the article option was selected. In addition, in the selection fields, the options Article Title, Abstract, and Keywords were chosen.

For Cochrane, the date of publication and the design of the article “Trial” were used as filters.

### 2.4. Eligibility Criteria

The criteria for the selection of the studies were the following:

- RCTs published in English and Spanish in the last ten years
- RCTs developed with an adult population (>18 years old) with balance disorders as a consequence of suffering a stroke in the previous six months before therapeutic intervention
- Studies that based their interventions on physiotherapeutic treatments using immersive or non-immersive virtual reality in isolation or compared to other forms of physiotherapeutic treatment
- RCTs with scores equal to or greater than 6 on the PEDro scale, in order to improve the quality of the review

### 2.5. Variables/Outcomes

The main variable considered in this review is balance, which can be considered as comprising two forms: static and dynamic.

The measurement instruments used in this review are those described in the clinical practice guideline by Veerbeek et al. [31]. These are included within the recommendations for outcome measures of the Academy of Neurologic Physical Therapy (ANPT) [32] therefore, we used the Berg scale (BBS) [17,24,33,34,35,36], and a functional reach test [17,33], as tools for measuring static equilibrium, and timed up and go test (TUG) [17,33,34,35,37,38,39] and the 10 m walking test [24,35,40] to assess dynamic balance.

### 2.6. Assessment of the Methodological Quality of the Included Studies

The PEDro methodological quality assessment scale was applied to all the selected articles [41,42]. This scale consists of 11 criteria and provides statistical information about the internal validity (criteria 2–9) to ensure that the results are interpretable (criteria 10–11). According to this scale, studies are considered of “excellent” quality with a PEDro score of 9–10, studies from 6 to 8 are considered “good,” scores between 4 and 5 are considered to be of “fair” quality, and studies with a score below 4 are considered to be of “poor” quality [43].

This review of the quality of the studies was carried out by two independent evaluators, and in situations of a discrepancy, a third was consulted.

The Cochrane risk-of-bias tool was used to analyze the risk of bias.

### 2.7. Analysis of Data

The quantitative synthesis of the results (meta-analysis) was carried out by another two authors. Nine different meta-analyses were carried out, the first three for static balancing, the fourth to the seventh meta-analyses show the dynamic balance virtual reality and the eighth and ninth were for the follow-up of static and dynamic balance.

In the second, third, and eighth meta-analyses, we found great heterogeneity between studies, so we used a random-effects model. In the rest of the meta-analysis, the studies were found to be very homogeneous, so a fixed-effects model was used. In all cases, a corresponding forest plot is presented together with a description of the risk of bias of each study, assessed using the Cochrane risk-of-bias tool. The publication bias was estimated using a funnel plot in all cases, and, wherever possible, the Begg and Egger tests were also performed. In addition, a sensitivity analysis was performed whenever possible to estimate the degree of influence of each article included in each meta-analysis on the results of the said meta-analysis.

When it was not possible to combine the results of the studies via a meta-analysis, narrative and descriptive summaries were completed, and qualitative synthesis of them was performed.

The strength of the evidence was assessed by a Grading of Recommendations Assessment, Development and Evaluation (GRADE) for pain intensity, pain duration, and quality of life using the GRADE Pro/Guideline Development Tool.

## 3. Results 

**Figure 1:**
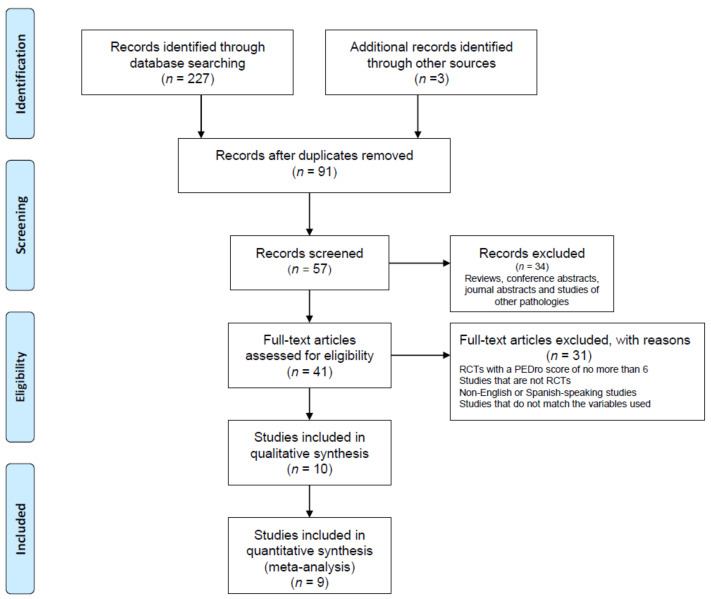
PRISMA flow diagram.

Through the searches, 227 studies were identified; however, after the elimination of duplicates and the application of the selection criteria, 10 studies remained, and 9 were included in meta-analyses that were performed (Figure 1).

The sociodemographic and clinical characteristics of the participants in each study are shown in Table 2. In relation to the characteristics of the population, the use of the Mini-Mental Test Exam to determine the cognitive capacity of patients is certified. We found differences in terms of the cut-off scores for including subjects in the studies (Table 2). In the studies by Kim et al. [38] and Cho et al. [17], the score was 24. In the studies presented by Lloréns et al. [20,24], the score used was 23, and in the studies by Lee et al. [34] and Park et al. [35], it was 21.

The mean age of the participants was 59.73 years in the experimental groups and 60.35 in the control groups. The size of the sample varied from one study to another, showing a mean of 32; the largest was 73 participants [37], and the smallest was 20 [24,35,39,40] (Table 2), but we noted that most of them had small sample sizes.

Regarding the number of sessions of virtual reality in the experimental group, we observed an average of 18.2 sessions (18.2 ± 7.146); the studies by Cho et al. [17] and Yom et al. [39] applied the most sessions, with a total of 30, and the trials from Bergman et al. [40], Lee et al. [34], Park et al. [35], and Kim et al. [38] applied the fewest, with 12 sessions each. On the other hand, in the control group, the average number of conventional physiotherapy sessions was 19, the lowest number of sessions occurred in the study by Kim et al. [38] with 8, and the highest number of sessions were scheduled in the studies by Cho et al. [17] and Yom et al. [39] with 30 sessions each.

There was agreement among the reviewers in the evaluation of the methodological quality of the studies, and the scores are shown in Table 3. All the articles included showed good quality, with scores between 6 and 8.

The least well-accomplished items in all the studies included were item 5 (the masking of participants) and item 6 (the masking of therapists). However, three items were not accomplished for at least two studies; Lee et al. [34] and Yom et al. [39] did not follow item 2, Bergmann et al. [40] and Park et al. [35] did not include item 7, and item 8 (the results for all the participants who received treatment or were assigned to the control group or, failing that, results that were analyzed by “intention to treat”) was not considered in six of the studies [17,33,35,38,39,40].

**Figure 2:**
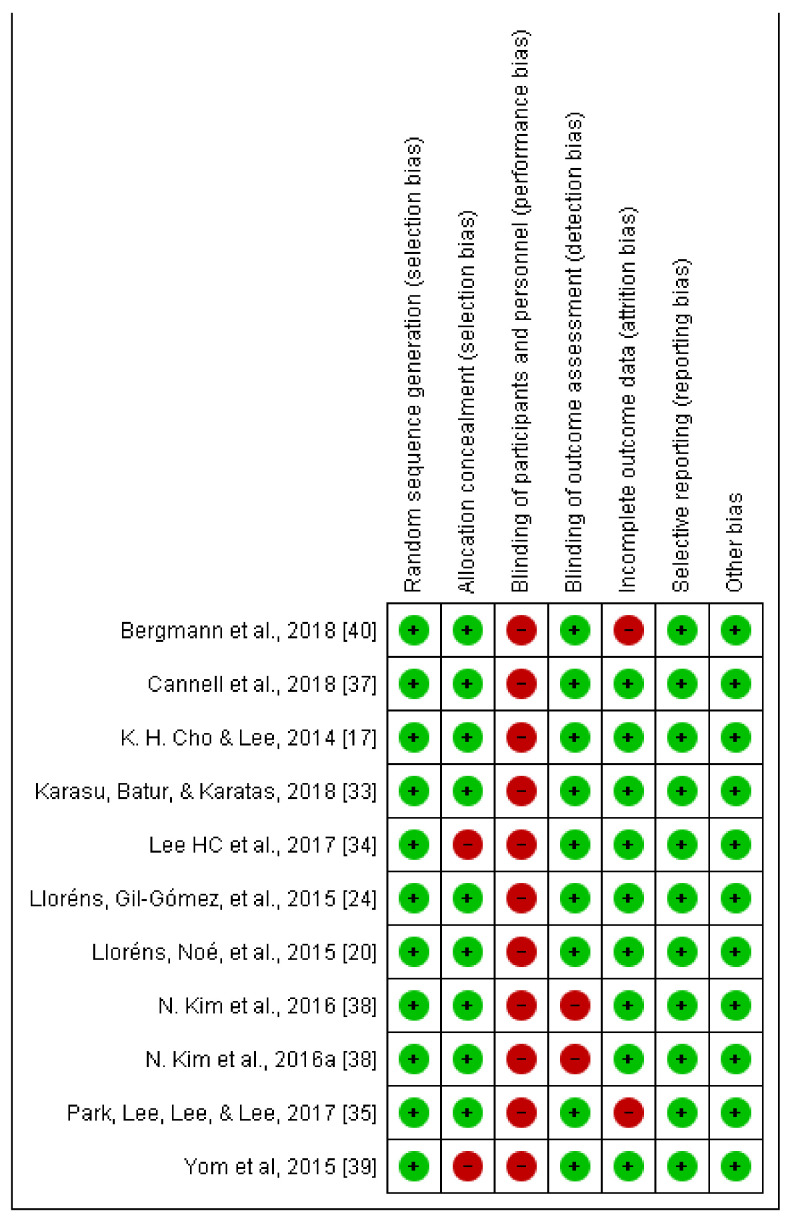
The Cochrane risk-of-bias.

Regarding the risk of bias, the Cochrane risk-of-bias tool was used, obtaining the results shown in Figure 2.

These data are aligned with what was obtained on the PEDro scale, and no study masked either the subjects or therapists.

Most of the studies included non-immersive virtual reality as an intervention through the use of video games (Table 2), while the studies by Kim et al. [38] and Cho et al. [17] focused on immersive virtual reality, using digital environments, not real ones, and therapeutic tools based on immersive virtual reality with real environments, respectively (Table 2).

Table 2 also shows how all the studies based their interventions on physiotherapeutic treatments associated with the use of virtual reality for the treatment of balance, compared with other forms of physiotherapeutic treatment, except the study by Llorens et al. [20], which compared to home virtual reality.

The times of the application of the interventions varied between four and eight weeks in all cases. However, the medium-term follow-up was only measured in four studies, and the measurements were performed differently, varying between six weeks and six months after the completion of the treatment sessions.

The outcomes for static and dynamic balance in all the studies included were collected; some of them assessed static, others dynamic and three of them collected data on both types of balance [17,34,38].

Subsequently, we classified the studies into two groups according to the type of intervention applied in the experimental group—that is, the type of virtual reality applied, immersive and non-immersive virtual reality.

Once these two groups were defined, the main variables of this review, static and dynamic balance, were taken into account to perform the segmentation of the subgroups (Table 4).

Static balance was evaluated using the Berg scale in six studies (five of applied non-immersive virtual reality and one of immersive) and the functional reach test in three studies (all of which applied non-immersive virtual reality).

To evaluate dynamic balance, we relied on two studies that used immersive virtual reality using the timed up and go test [17,38]; in addition, we consulted six studies that applied non-immersive virtual reality, three of which used the 10 m test [24,35,40], while Lee et al. [34] Karasu et al. [33], and Yom et al. [39] used the timed up and go test.

### 3.1. Static Balance

The static balance was measured in seven of the ten studies included.

In relation to non-immersive virtual reality, as shown in Table 4, and as a result of the qualitative analysis, results in favor of non-immersive virtual reality in static equilibrium were observed in four of the studies [20,24,34,35]. Although improvements were presented in the other three studies [33,34,37], they were not significant. In the case of the immersive virtual reality application, we found optimal results with significant improvements in a single study by Cho et al. [17], measured using the Berg scale. In light of these results, we consider that there is a trend in favor of virtual reality treatment, whether immersive or non-immersive, for improving static balance, as measured with the Berg scale.

**Figure 3:**
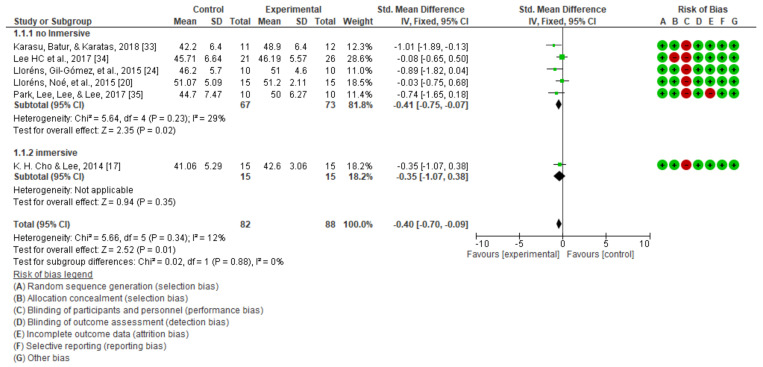
Static balance as measured using the Berg scale.

**Figure 4:**
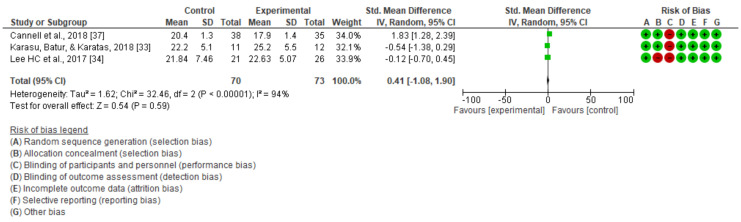
Static balance as measured using the functional reach test.

**Figure 5:**
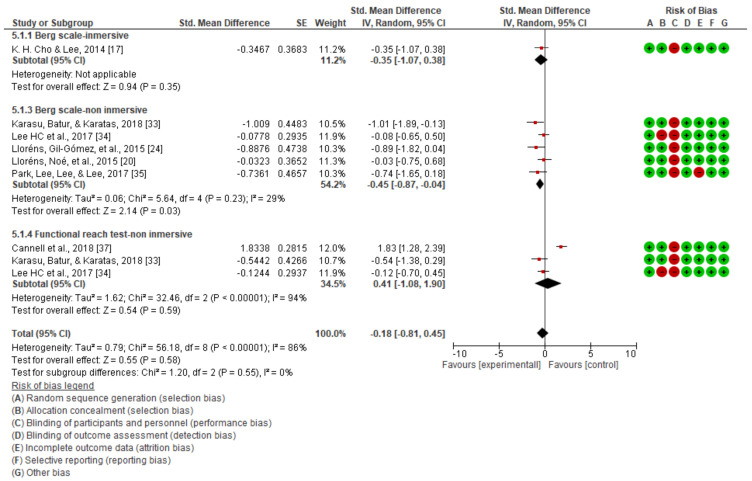
Global results for static balance.

The results obtained in the quantitative synthesis and meta-analysis carried out for static balance are shown in Figure 3, Figure 4 and Figure 5.

A significant improvement in static balance, evaluated with the Berg scale, was observed with the applied treatment in the comparison of the post-intervention scores (mean difference (MD) = −1.97; 95% CI = −3.51 to −0.44; standardized mean difference (SMD) = −0.40; 95% CI = −0.70 to −0.09). Figure 3 also shows that the study showing a greater impact of virtual reality on static balance was that using the Berg scale and performed by Karasu et al. [33], who found the largest mean difference between groups. When performing the analysis by the type of intervention in the case of the “non-immersive virtual reality,” there was a statistically significant difference in favor of the experimental group, which was not the case in the only study included in the immersive group.

However, with the functional reach test, there was no significant improvement with the applied treatment in comparison with the post-intervention results (MD = 0.06; 95% CI = −3.47 to 3.59; SMD = 0.41 95%; CI = –1.08 to 1.90) (Figure 4).

Analyzing the static equilibrium as a global outcome (Figure 5), although there seems to be a tendency in favor of the experimental intervention in the descriptive analysis, we did not find that this improvement was significant in the meta-analysis (SMD: −0.18; CI: –0.81 to 0.45).

### 3.2. Dynamic Balance

Dynamic balance was evaluated in seven of the ten included studies.

From the qualitative analysis shown in Table 2, we observed significant changes favorable to the experimental group in the studies carried out with non-immersive virtual reality by Karasu et al., Lee et al., Park et al., and Yom et al. [33,34,35,39], and in the two studies conducted using immersive virtual reality by Kim et al. [38] and Cho et al. [17].

**Figure 6:**
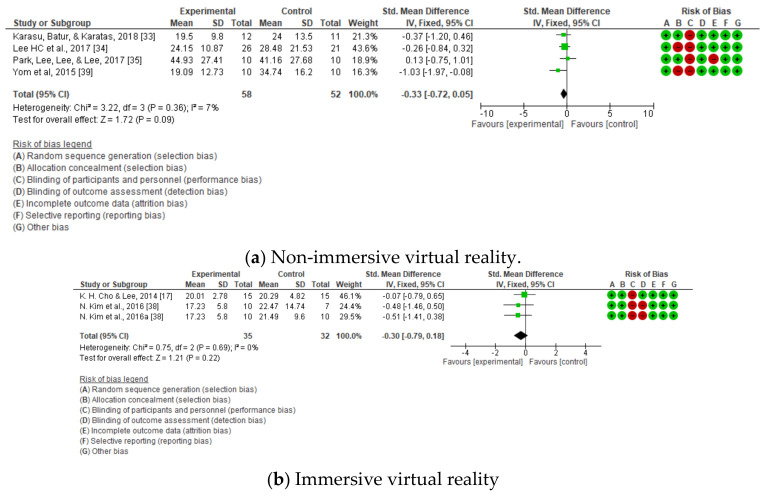
Dynamic balance as measured using the timed up and go test. (**a**) Non-immersive virtual (**b**) Immersive virtual reality.

**Figure 7:**
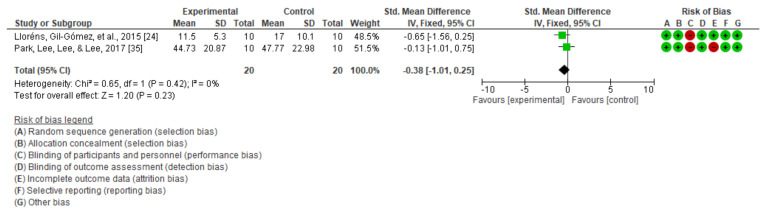
Dynamic balance as measured using the 10 m test.

**Figure 8:**
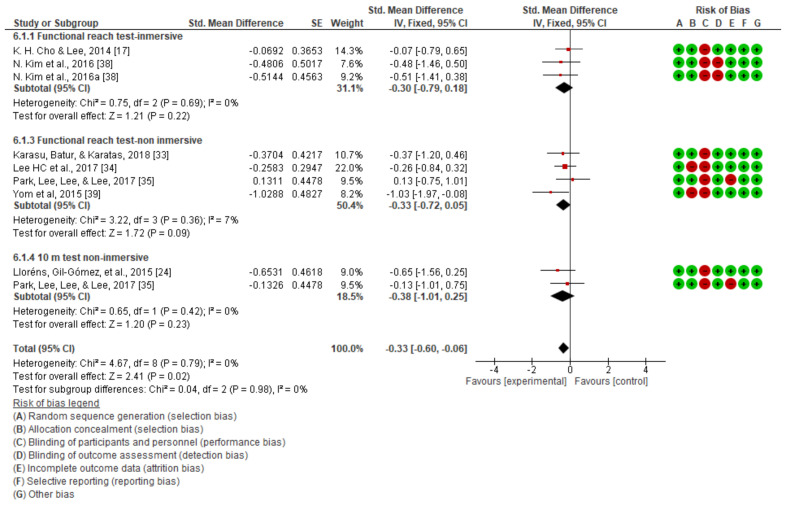
Global dynamic balance.

The results of the quantitative synthesis and meta-analysis carried out for dynamic balance are shown in Figure 6, Figure 7 and Figure 8.

Upon observing the four forest plots, we can see that, in terms of global balance, there was a significant improvement in favor of the experimental group when comparing the post-intervention measurements (standard mean difference (SMD) = −0.33; 95% CI = −0.6 to −0.06), without differentiating between immersive and non-immersive virtual reality (Figure 8).

On the other hand, upon analyzing the dynamic balance through the timed up and go test, in a non-immersive intervention (SMD = −0.30; 95% CI = −0.79, 0.18; MD = −1.06; CI = −3.60, 1.49) (Figure 6a) and an immersive one (SMD = −0.33; 95% CI = −0.72, 0.05; MD = −6.36; CI = −12.31, −0.41) (Figure 6b), as well as with the 10 m walking test (SMD = −0.38; 95% CI = −1.01, 0.25; MD = −5.21; IC = −11.84, 1.43) (Figure 7), in its only non-immersive modality, we found that there were no significant differences.

Regarding the follow-up of the results, only three studies carried out such follow-up in terms of the static equilibrium and non-immersive intervention, at eight weeks for Karasu et al. [33], twelve weeks for Lloréns et al. [20], and six months for Lee et al. [34].

**Figure 9:**
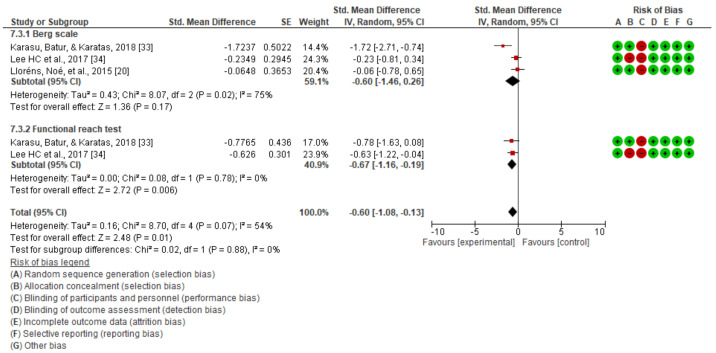
Meta-analysis of follow-up on static balance.

In Figure 9, the improvement in this variable is shown to be statistically significant, with an SMD of −0.6 and 95% CI of −1.08 to −0.13 globally, and it was also significant in the case of measures with the functional reach test, with an SMD of −0.67 and 95% CI of −1.16 to −0.19.

**Figure 10:**
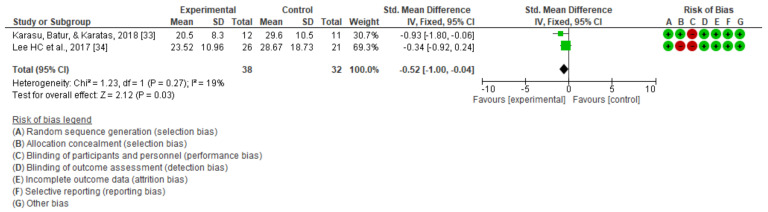
Meta-analysis of follow-up on dynamic balance.

Karasu et al. [33] and Lee et al. [34], also carried out follow-up for the dynamic balance variable measured with the timed up and go test at eight weeks and six months, respectively, for non-immersive virtual reality. Figure 10 shows a slight but significant improvement in this dynamic balance (SMD = −0.52; 95% CI = −1.00 to −0.04; MD = −7.42; CI = −13.32, −1.52).

**Figure 11:**
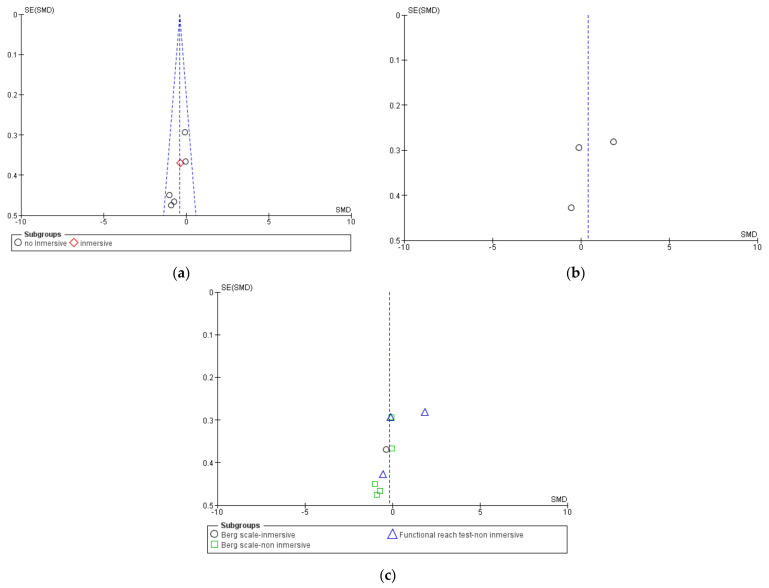
Funnel plots for static balance. (**a**) Static balance measured using Berg scale, (**b**) static balance measured using functional reach test, and (**c**) global static balance.

**Figure 12:**
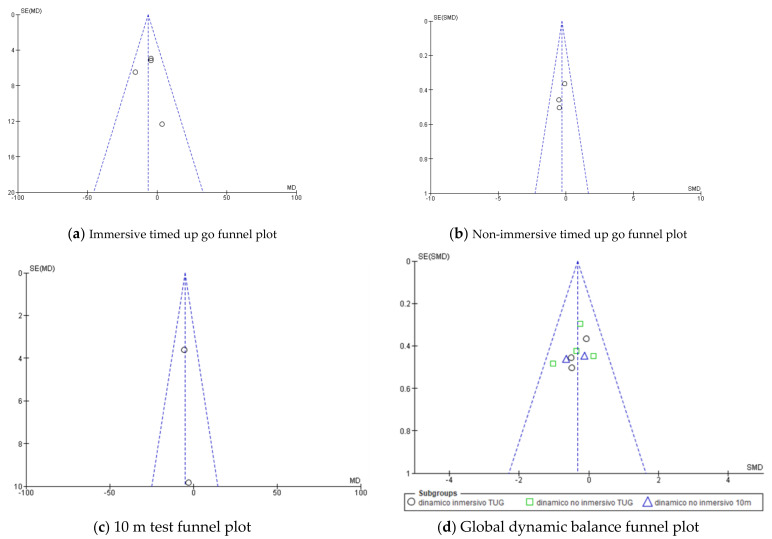
Funnel plots for dynamic balance. (**a**) Immersive timed up and go funnel plot, (**b**) non-immersive timed up and go funnel plot, (**c**) 10 m test funnel plot, and (**d**) global balance funnel plot.

**Figure 13:**
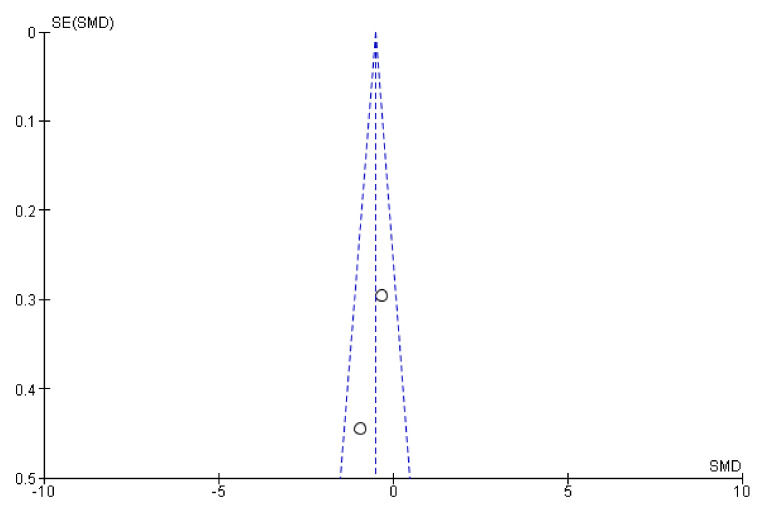
Funnel plot for dynamic balance follow-up.

When possible, Begg and Egger tests were carried out to analyze the existence of publication bias. There was no statistical evidence of the existence of publication bias, according to the results of these tests (*p* > 0.05). This finding is corroborated by the funnel plots (Figure 11, Figure 12 and Figure 13). The sensitivity analysis indicated that the overall results from these meta-analyses were not substantially modified by the elimination of any result.

## 4. Discussion

Following the systematic review and meta-analysis of the studies included in this review, the use of immersive and non-immersive virtual reality for the treatment of balance appears to be effective for global dynamic balance, although not for global static balance. However, for studies that follow stroke patients, it is effective for the treatment of both static and dynamic balance.

The studies have been classified according to the level of evidence and the assignment of the grade or strength of recommendation (GRADE). For dynamic balance, a high grade of recommendation has been assigned, which shows that there is high confidence in the coincidence between the real and estimated effect. For the cases in which there is follow-up, the grade of recommendation is moderate as for the static global balance. (Table 5).

The methodological quality of the studies is good, taking into account that, in this review, only those articles with scores higher than 6 on the PEDro scale were included. A common situation was observed in all of them. Neither the subjects nor the therapists who administered the therapy were blinded, which is understandable given the difficulty of blinding in this intervention as is often the case with other physiotherapeutic interventions.

According to the studies analyzed, the Mini-Mental Test scale is used as an inclusion criterion to determine whether the cognitive levels of the subjects are optimal for understanding and executing the intervention [17,20,25,33,35,38,44].

Clinical interventions adopted for balance rehabilitation are usually based on the principles of neuroplasticity and motor learning. In order to improve sensory and motor skills, therapeutic exercises oriented to different tasks are applied [12,45].

In addition, these proposed tasks are adjusted to balance work through specific therapeutic exercises generally oriented to the proposed objectives, as is the case of the studies examined in this review for balance work.

All the studies have supported balance work in a specific way with physiotherapy through therapeutic exercises applied conventionally or carried out through virtual reality.

Virtual reality, designed with computer hardware and software, uses interactive simulations. They are created to present users with the opportunity to participate in environments that simulate real-world situations and events [46,47], but in a safe environment (clinics, hospitals, etc.). It is noted that most of the studies published in the last ten years have used virtual reality in the treatment of balance in people with neurological problems.

In the experimental group, most studies combined virtual reality with conventional physiotherapy exercises [25,33,35,37,39,40]. The relevance of combining with other types of exercises for the treatment of balance is understandable since in neurological processes not only balance is affected but also functional capacity is impaired.

In the studies that used non-immersive virtual reality, we observed that a multitude of digital platforms were used, generally linked to commercial brands related to entertainment such as Wii [33,48] and Xbox [33,38].

This situation led to a diversity of interventions in the groups. Most used virtual reality devices in controlled experimental settings, while Lloréns et al. [20] applied the virtual reality intervention in subjects’ homes in isolation, aligning with Gallagher et al. [49], who stated that home-based interventions allow greater adherence to treatments.

As for immersive virtual reality devices intended to be used for therapeutic purposes in the field of neurological disorders [50], they allow simulating environments in controlled laboratory conditions with a very high level of interactivity.

However, the use of these devices is scarce in the research setting. Perhaps, due to the state of the technology and its high cost, which has hindered access to users. This could be the reason, we only found two studies in this review with this type of virtual reality, associated with conventional physiotherapy exercises [17,38].

The technological situation and increased competition within the market may make these devices more accessible to organizations and users. Therefore, in the future, we could see more immersive reality devices being used in different areas, including medicine [51,52].

If we focus on the treatment for the patients in the control group, it is observed that in most cases, conventional physiotherapy exercises were prescribed, whose efficacy has already been demonstrated [53,54] to improve balance in the population included in this review. As in the study by Cannell et al. [37], in which conventional physical therapy was combined with a protocol of functional exercises for balance, strength, and/or endurance, they have also shown satisfactory results for these variables [55,56].

Focusing on the measurement instruments, we found an inconsistency in relation to the Berg scale. One study used it to measure dynamic balance [17], although in our study, and following the premises of the evidence and the recommendations of international clinical guidelines, this scale was included as a variable for measuring static balance [57,58].

Regarding the efficacy of the treatment on static balance, measured by means of the Berg scale, we found significant differences at a general level and, more specifically, for non-immersive virtual reality. No clinically relevant changes are observed since Alghadir et al. [59] established a score of 2.7 points for this change to occur. In particular, we have found that the articles by Karasu et al. [33], HC Lee et al. [34], and Cannell et al. [37], which use the functional reach test to assess static balance do not present significant changes. Therefore, we believe that virtual reality treatment in this type of balance should incorporate functional reaching scenarios to improve the results in static balance.

In the case of dynamic balance, measured through the timed up and down test in the studies using virtual reality, no significant improvements were found. However, a clinically relevant change of 6.36 s. was found, as Alghadir et al. [59], point out that the minimum change detectable is 3.2 s. For the 10 m test, no significant improvements were found.

The extrapolated results of the overall dynamic balance analysis indicated significant changes in the quantitative analysis, favorable to the experimental group, which may be due to the increased sample size when incorporating all studies [25,33,34,35,38,39].

Regarding patient follow-up over time and focusing on static equilibrium, three studies followed up at eight weeks [33], twelve weeks [20], and six months [34]. Follow-up for dynamic balance occurred in two studies [33,34]. In all of them, the interventions used non-immersive virtual reality and significant improvements were obtained for both balances.

Medium-and long-term follow-up of the subject is necessary to study the effect of treatment on outcomes over time, due to the importance of rehabilitation in terms of neural plasticity and motor relearning after brain damage [60].

Regarding the limitations, it is worth highlighting the heterogeneity found in terms of the measurement instruments for the variables, as well as the interventions, which makes it difficult to compare data and establish solid conclusions.

## 5. Conclusions

We can conclude that the application of physiotherapy through the use of virtual reality, in a combined or isolated manner, for patients who have suffered strokes seems to be beneficial for static (measured by Berg scale) and dynamic balance as a global outcome. It also appears to be effective for maintaining the medium- and long-term results measured globally.

However, more studies of good methodological quality are needed, with larger sample sizes and unified instruments for measuring equilibrium, to corroborate these conclusions.

## Figures and Tables

**Table 1:** Database search strategies.

	MEDLINE	PEDRO	COCHRANE	SCIELO	SCOPUS
Boolean Osperator	AND/OR	AND/OR	AND/OR	AND/OR	AND/OR
Keywords	“Physiotherapy “or “Physical Therapy” “virtual Reality”, “Immersive Virtual Reality” or “Non Immersive Virtual Reality” “Stroke” “Balance” “static balance” “Dynamic balance”	“Physiotherapy “or “Physical Therapy” “virtual Reality”, “Immersive Virtual Reality” or “Non Immersive Virtual Reality” “Stroke” “Balance” “static balance” “Dynamic balance”	“Physiotherapy “or “Physical Therapy” “virtual Reality”, “Immersive Virtual Reality” or “Non Immersive Virtual Reality” “Stroke” “Balance” “static balance” “Dynamic balance”	“Physiotherapy “or “Physical Therapy” “virtual Reality”, “Immersive Virtual Reality” or “Non Immersive Virtual Reality” “Stroke” “Balance” “static balance” “Dynamic balance”	“Physiotherapy “or “Physical Therapy” “virtual Reality”, “Immersive Virtual Reality” or “Non Immersive Virtual Reality” “Stroke” “Balance” “static balance” “Dynamic balance”
Filters	“Article type” (RCT y clinical trial protocol) “Publication date” (last 10 years) species (humans) “languages” (English and Spanish)	“Subdisciplina”, Neurology “Method “, clinical trial “Published since”, 2010	“Publication date”, 2010 “Type of article”, Trial	“Database”, was selected Article “Languages”. Spanish and English “Year of publication”, from 2010 to 2020	“Time range of search”, from the year 2010 to the present. “Document type”, the article option was selected. “Selection fields”, the option was selected. Article title, Abstract y Keywords.

**Table 2:** Studies included in the review.

STUDY	POPULATION AND CHARACTERISTICS	INTERVENTION	TYPE OF VIRTUAL REALITY	COMPARISON	EVALUATION/FOLLOW-UP	MEASURING INSTRUMENTS
Karasu et al., 2018 [33]	23 patients GE (*n* = 12): 62.3 ± 11.79 GC (*n* = 11): 64.1 ± 12.2 Ability to understand and follow simple commands	20 sessions: conventional neurological rehabilitation + virtual reality (Wii)	Nonimmersive	Conventional neurological rehabilitation 20 sessions: 5 sessions of 2–3 h/week over 8 weeks	Static and dynamic balance assessment at baseline and 4 weeks. Follow-up at 8 weeks	Berg balance scale Functional reach test Postural assessment scale for stroke patients Timed up and go testS tatic balance index
Bergmann et al., 2018 [40]	20 patients GE (*n* = 10): 62 ± 11 GC (*n* = 10): 65 ± 8 Inability to ambulate without help or assistance from another person (functional ambulation rating ≤2), Cognitive abilities to understand and follow simple verbal instructions.	Physiotherapy + virtual reality 8 sessions of physiotherapy + 12 sessions of virtual reality	Non-immersive	Physiotherapy + Lokomat 8 sessions of physiotherapy + 12 sessions of Lokomat	Assessment of dynamic balance at baseline and 4 weeks. Follow-up of dynamic balance at 8 weeks	Questionnaire IMI Functional ambulation classification 10 m walking test 6 min walking test Medical Research Council
Lee et al., 2017 [34]	50 patients GE (*n* = 26): 59.35 ± 8.95 GC (*n* = 24): 55.76 ± 9.59 Ability to understand game instructions Ability to stand for 15 min	Conventional physiotherapy + virtual reality (kinetic sports), 12 sessions	Non-immersive	Conventional physiotherapy + balance exercise protocol 12 sessions	Static and dynamic balance assessment at baseline and 6 weeks Follow-up at 6 months	Berg balance scale Functional scope test Timed up and go test Barthel scale modified ABC specific test of balance stroke impact scale
Park et al., 2017 [35]	20 patients GE (*n* = 10): 62.00 ± 17.14 GC (*n* = 10): 65.30 ± 10.51 Minimum score of 21 on mini-mental test Ability to walk 10 m with or without assistance	Conventional physiotherapy + virtual reality (Xbox), 12 sessions	Non-immersive	Conventional physiotherapy 12 sessions	Assessment of static and dynamic balance at baseline and 6 weeks No follow-up	Fugl–Meyer assessment Berg balance scale Timed up and go 10 m walking test
Lloréns, Noé, et al., 2015 [20]	30 patients GE (*n* = 15): 55.47 ± 9.63 GC (*n* = 15): 55.60 ± 7.29 On the Brunel scale (Section 3), levels 7–12 More than 23 points in the mini-mental test	Virtual reality at home (Kinect) 20 sessions	Non-immersive	Virtual reality in the clinic 20 sessions	Static balance evaluation at baseline and 8 weeks Follow-up of static balance at 12 weeks	Berg balance scale Tinetti scale Brunel balance assessment Performance-oriented mobility assessment System usability scale Intrinsic motivation inventory
Lloréns et al., 2015 [24]	20 patients GE (*n* = 10): 58.3 ± 11.6 GC (*n* = 10): 55 ± 11.6 More than 23 points in the mini-mental test Ability to remain in a standing position without assistance (Section 3, level 7 Brunel scale).	Conventional physiotherapy + virtual reality (virtual rehabilitation system), 20 sessions	Non-immersive	Conventional physiotherapy 20 sessions	Evaluation of static and dynamic balance at baseline and 4 weeks No follow-up	Berg balance scale Tinetti scale Brunel balance assessment 10 m walking test
Cho et al., 2014 [17]	30 patients GE (*n* = 15): 63.53 ± 5.54 years GC (*n* = 15): 65.86 ± 5.73 years Ability to walk 10 m with or without assistance Ability to understand simple instructions (>24 on the mini mental test)	Conventional rehabilitation + immersive virtual reality (treadmill) 30 sessions	Immersive	Conventional physiotherapy 30 sessions	Static and dynamic balance assessment at baseline and 6 weeks No follow-up	Berg balance scale Timed up and go test Platform for postural and gait control
Cannell et al., 2018 [37]	73 patients GE (*n* = 35): 72.8 ± 10.4 years GC (*n* = 38): 74.8 ± 11.9 years Ability to follow instructions and communicate with researchers	Conventional physiotherapy + virtual reality (Jintronix Rehabilitation System^TM^) 14 sessions	Non-immersive	Conventional physiotherapy + functional exercise protocol, strength, balance, and endurance (14 sessions)	Evaluation of static and dynamic balance at baseline and 8 weeks or at hospital discharge. No follow-up	Functional reach test Functional independence measure (FIM) Timed up and go test
Kim et al., 2016 [38]	30 patients GVRCA (*n* = 10): 56.20 ± 7.56 years GCA (*n* = 10): 52.00 ± 7.27 years GC (*n* = 7): 48.71 ± 9.27 years Ability to walk 6 m without technical assistance More than 24 points in the mini mental test	GVRCA: conventional physiotherapy (8 sessions) + virtual reality on treadmill (12 sessions)	Immersive	1-GCA: conventional physiotherapy (8 sessions) + walking in real environments (12 sessions) 2-GC: conventional physiotherapy: 8 sessions	Evaluation of dynamic balance at baseline and 4 weeks No follow-up	Timed up and go test ABC Scale 6 min walking test
Yom et al., 2015 [39]	20 patients GE (*n* = 10): 64.60 years GC (*n* = 10): 78.10 years Score greater than 24 on the mini mental test	Conventional physiotherapy (previous) + virtual reality ankle exercises (30 sessions)	Non-immersive	Conventional physiotherapy (previous) + video observation of the same exercises (30 sessions)	Dynamic baseline evaluation at baseline and 6 weeks No follow-up	Timed up and go test Modified Ashworth Tardieu Scale GAITRite computerized evaluation system

**Table 3:** PEDro score.

Trial	Subjects Were Randomly Allocated to Groups (in a Crossover Study, Subjects Were Randomly Allocated an Order in Which Treatments Were Received).	Alloction Was Concealed.	The Groups Were Similar at Base-Line Regarding the Most Important PrognosticIndicators	There Was Blinding of All Subjects.	There Was Blinding of All Therapists Who Administered the Therapy.	There Was Blinding of All Assessors Who Measured at Least One Key Outcome.	Measures of at Least One Key Outcome Were Obtained from more than 85% of the Subjects Initially Allocated to Groups.	All Subjects for Whom Outcome Measures Were Available Received the Treatment or Control Condition as Allocated, or, Where This Was Not the Case, Data for at Least One Key Outcome Were Analyzed by “Intention to Treat”.	The Results of between-Group Statistical Comparisons Are Reported for at Least One Key Outcome.	The Study Provides Both Point Measures and Measures of Variability for at Least One Key Outcome.	Total Score PEDro Scale
Karasu et al. [33]	Yes	Yes	Yes	No	No	Yes	Yes	No	Yes	Yes	8
Bergmann et al. [40]	Yes	Yes	Yes	No	No	Yes	No	No	Yes	Yes	7
Lee et al. [34]	Yes	No	Yes	No	No	Yes	Yes	Yes	Yes	Yes	8
Park et al. [35]	Yes	Yes	Yes	No	No	Yes	No	No	Yes	Yes	7
Lloréns et al. [24]	Yes	Yes	Yes	No	No	Yes	Yes	Yes	Yes	Yes	8
Lloréns et al. [20]	Yes	Yes	Yes	No	No	Yes	Yes	Yes	Yes	Yes	8
Cho et al. [17]	Yes	Yes	Yes	No	No	Yes	Yes	No	Yes	Yes	7
Cannell et al. [37]	Yes	Yes	Yes	No	No	Yes	Yes	Yes	Yes	Yes	8
Kim et al. [38]	Yes	Yes	Yes	No	No	Yes	Yes	No	Yes	Yes	7
Yom et al. [39]	Yes	No	Yes	No	No	Yes	Yes	No	Yes	Yes	6

**Table 4:** Grouping of studies according to the type of virtual reality and the balance measured.

	VARIABLES	ASSESSMENT INSTRUMENTS	STUDY	RESULTS
NON-IMMERSIVE VIRTUAL REALITY	STATIC BALANCE	Berg scale	Karasu et al. [33]	No significant differences between groups in terms of primary and secondary outcome measures at admission (*p* > 0.05).
			H. C. Lee et al. [34]	Significant improvements on Berg’s scale (*p* = 0.000).
			Park et al. [35]	Significant improvements in the experimental group on the Berg scale (*p* < 0.05).
			Lloréns, Gil-Gómez, et al. [24]	Significant improvement in both groups in terms of balance (*p* = 0.006).
			Lloréns, Noé, et al. [20]	Significant improvements on the Berg scale (*p* < 0.05) in the experimental group.
		Functional reach test	Karasu et al. [33]	No significant differences between groups in terms of primary and secondary outcome measures at admission (*p* > 0.05).
			H. C. Lee et al. [34]	No significant changes were observed.
			Cannell et al. [37]	No significant changes were found between the two groups.
	DYNAMIC BALANCE	Timed up and go test	Karasu et al. [33]	No significant differences between groups on primary and secondary outcomes at entry (*p* > 0.05).
			H. C. Lee et al. [34]	Significant improvements in the TUG scale (*p* = 0.005.).
			Park et al. [35]	Significant improvements in the experimental group for the timed up and go test (*p* < 0.05).
			Yom et al. [39]	Significant improvements in dynamic balance (*p* < 0.05).
		10 m walking test	Bergmann et al. [40]	Significant improvements in both groups in terms of walking speed (*p* < 0.01).
			Park et al. [35]	Significant improvements in the experimental group in the timed up and go and 10 m tests p < 0.05).
			Lloréns, Gil-Gómez, et al. [24]	Significant improvement in both groups in terms of gait (*p* = 0.001).
IMMERSIVE VIRTUAL REALITY	STATIC BALANCE	Berg scale	K. H. Cho & Lee. [17]	There were significant improvements in terms of static balance (*p* < 0.01).
		Functional reach test		
	DYNAMIC BALANCE	Timed up and go test	K. H. Cho & Lee. [17]	Significant improvements in both groups in terms of dynamic balance and gait (*p* < 0.05).
			Kim et al. [38]	Significant changes in gait speed in each group (*p* < 0.01).
		10 m walking test		

**Table 5:** Recommendations according to GRADE tool.

Certainly Assessment							№ of Patients		Effect		Certainly	Importance
№ of Studies	Study Design	Risk of Bias	Inconsistency	Indirectness	Imprecision	Other Considerations	Virtual Reality	Other	Relative (95% CI)	Absolute (95% CI)		
Static balance												
7	Randomized trials	Not serious	Not serious	Not serious	serious	None	161	152	-	SMD −**0.18** (−0.81 to 0.45)	⨁⨁⨁◯ MODERATE	NOT IMPORTANT
Dynamic balance												
7	Randomized trials	Not serious	Not serious	Not serious	Not serious	None	123	114	-	SMD −**0.33** (−0.6 a −0.06)	⨁⨁⨁⨁ HIGH	NOT IMPORTANT
Follow-up of Static balance												
3	Randomized trials	Not serious	Not serious	Not serious	serious	None	53	47	-	SMD −**0.6** (−1.08 to −0.13)	⨁⨁⨁◯ MODERATE	NOT IMPORTANT
Follow-up of dynamic balance												
2	Randomized trials	Not serious	Not serious	Not serious	serious	None	38	33	-	SMD −**0.52** (−1 a −0.04)	⨁⨁⨁◯ MODERATE	NOT IMPORTANT

CI: Confidence interval; SMD: Standard mean difference; MD: Mean difference; This symbol is from the GRADE tool, appears based on the strength of the evidence, more ⨁ means stronger evidence, that is why 4 ⨁ is high and 3 ⨁ is moderate.

## Data Availability

Not applicable.
